# Emphysematous Cystitis With Extraperitoneal Bladder Perforation in a Frail Elderly Patient on Long-Term Corticosteroid Therapy Without Diabetes: A Hidden Geriatric Threat in a Frail Elderly Patient

**DOI:** 10.7759/cureus.110753

**Published:** 2026-06-12

**Authors:** Babajide F Ajeyomi, Elba B Peter, Friday Nehikhare, Karunayokiny Dineshkumar, Baazla F Kakroo

**Affiliations:** 1 General Medicine/Geriatrics, Aneurin Bevan University Health Board, Newport, GBR; 2 Medicine, Aneurin Bevan University Health Board, Newport, GBR; 3 General Medicine/Geriatric Medicine, Royal Gwent Hospital, Newport, GBR; 4 Care of the Elderly, Anuerin Bevan University Health Board, Newport, GBR; 5 Geriatrics, Royal Gwent Hospital, Newport, GBR

**Keywords:** emphysematous cystitis(ec), emphysematous urinary tract infection, urinary bladder diseases, urinary bladder perforation, urinary tract infection in elderly

## Abstract

Emphysematous cystitis is a rare but serious urinary tract infection characterized by gas formation within and around the bladder wall, affecting elderly immunocompromised patients. The case report describes an 82-year-old frail gentleman admitted to a geriatric ward with nonspecific symptoms, including malaise, later diagnosed with emphysematous cystitis complicated by bladder perforation. The case is unusual due to the absence of classic urinary symptoms, chronically elevated C-reactive protein (CRP) with emphysematous cystitis presenting insidiously in a frail elderly patient on long-term high-dose corticosteroids. Diabetes mellitus is the most frequently reported risk factor; however, other forms of immunosuppression remain under-recognised. Management involved a multidisciplinary team approach with early antibiotic therapy, urology and microbiology input, and careful monitoring, resulting in improvement and resolution of bladder pathology. This case highlights the importance of maintaining a broad differential diagnosis in frail older adults with multiple comorbidities and immunosuppression and reinforces the importance of timely diagnostics and comprehensive geriatric assessments.

## Introduction

Emphysematous cystitis is a rare but serious infection of the bladder characterised by gas formation within the bladder wall and lumen, with reported mortality rates ranging from 7% to 14% [1]. It predominantly affects older adults, with a mean age of approximately 66 years [2], and is most commonly associated with risk factors such as diabetes mellitus, neurogenic bladder, urinary tract obstruction, chronic catheterisation, immunosuppression, and recurrent urinary tract infections. The condition is typically caused by gas-forming organisms, most frequently *Escherichia coli *and *Klebsiella pneumoniae *[3]. Clinical presentation is variable and often nonspecific, ranging from mild urinary symptoms such as dysuria and haematuria to severe systemic illness, which can lead to delayed or missed diagnosis. Diagnosis is primarily radiological, with computed tomography, with or without contrast, representing the most sensitive imaging modality for detecting intramural and intraluminal gas and outperforming plain abdominal X-ray and ultrasound [4]. Prompt recognition and early management with antimicrobial therapy, bladder drainage, and urological involvement are essential to prevent serious complications, including bladder perforation and systemic sepsis.

This report presents an unusual case of emphysematous cystitis with bladder perforation occurring in a frail 82-year-old man, whose primary risk factor was long-term high-dose corticosteroid therapy. A key educational focus is the role of a chronically elevated and fluctuating CRP in the context of known systemic vasculitis on long-term high-dose steroids, which rendered inflammatory markers unreliable as a discriminating infective signal and contributed to diagnostic delay.

## Case presentation

An 82-year-old man with a background of vasculitis and heavily immunosuppressed due to long-term high-dose corticosteroid therapy attended a neurology appointment with a four-week prior history of feeling unwell and tired intermittently, but no other complaints. Blood tests demonstrated a C-reactive protein (CRP) of 71 mg/L with ESR of 9 mm/hr. Critically, this result was interpreted in the context of a 14-month history of substantially and variably elevated CRP values, ranging from 18 to 203 mg/L, driven by his underlying vasculitic disease. The mildly elevated CRP at this visit was therefore not clearly distinguishable from his established inflammatory background, and the working differential at neurology follow-up included possible vasculitis reactivation, and oral prednisolone was increased accordingly.

At the second clinic visit, a follow-up after three weeks, the patient reported worsening mobility, weight loss, and increasing dependence on his wife for support. Repeat CRP had dropped to 59 mg/L. He was treated as a case of reactivation of vasculitic neuropathy with intravenous methylprednisolone 1 g daily for five days, as he couldn't tolerate mycophenolate mofetil alongside referral to physiotherapy. A CT scan of the thorax, abdomen, and pelvis was requested as an outpatient investigation to rule out any other cause of elevated CRP, as there was no clear symptom to suggest a focus of infection. The scan was performed three weeks after the request.

At the third clinic visit, a week after the last clinic visit, the patient was still feeling unwell. The neurologist maintained a high index of suspicion and screened for infection with bloods and urine microscopy, culture, and sensitivity (MCS) despite an unconvincing inflammatory marker whilst waiting for the report of CT thorax, abdomen, and pelvis. Urine MCS report was available after three days, which isolated *Klebsiella pneumoniae*, sensitive to pivmecillinam and trimethoprim but resistant to amoxicillin. Following receipt of the MCS result, attempts were made to contact the patient directly by telephone; however, the patient could not be reached. The neurologist subsequently liaised with the patient's general practice, and the general practitioner (GP) prescribed trimethoprim 200 mg twice daily for three days based on the neurologist's clinical direction. The neurologist also attempted to expedite reporting of the CT scan, as it had been performed but not reported.

Approximately six weeks after the initial radiological CT scan request, the CT thorax, abdomen, and pelvis report revealed significant air lining of the bladder walls consistent with emphysematous cystitis with possible bladder perforation and incidental finding of bilateral lower segment pulmonary emboli. The findings were promptly escalated, patient contacted, leading to urgent hospital admission.

Upon admission, the patient experienced shortness of breath, mild pleuritic chest pain on the left side, and ongoing functional decline. He did not have fever, poor appetite, abdominal pain, or low back pain, and no recent history of catheterisation. Two days into antibiotic treatment during hospitalisation, he developed mild haematuria with purulent discharge.

His past medical history included polymyalgia rheumatica, mononeuritis multiplex with bilateral foot drop and median and ulnar nerve palsies, and hypertension. Socially, he lived with his wife in a bungalow, was independent with a package of care, mobilised with a Zimmer frame, was an ex-smoker (having quit 20 years earlier), and did not consume alcohol.

On physical examination, he was alert, oriented to time, place, and person, afebrile, and not in respiratory or painful distress. Vital signs revealed heart rate 106 bpm, blood pressure 111/58 mmHg, respiratory rate 20 per minute, and oxygen saturation 80% on room air, prompting immediate commencement of supplemental oxygen. Heart sounds were normal. Abdominal examination was unremarkable with no tenderness, and no signs of deep vein thrombosis or lower limb oedema were noted.

Laboratory investigations, including inflammatory markers, full blood count, renal function, coagulation profile, bone and liver profile, urine microscopy, and microbiology, are summarised in Table 1.

**Table 1:** Laboratory findings on admission eGFR: estimated glomerular filtration rate; APTT: activated partial thromboplastin time; ALP: alkaline phosphatase; ALT: alanine aminotransferase; HPF: high-power field

Test Category	Parameter	Result	Normal Range
Inflammatory markers	C-reactive protein (CRP)	177 mg/L	< 5 mg/L
Hematology	White cell count	12.3 ×10⁹/L	4.0–11.0 ×10⁹/L
	Neutrophils	11.4 ×10⁹/L	2.0–7.5 ×10⁹/L
Renal function	Sodium	139 mmol/L	135–145 mmol/L
	Potassium	4.3 mmol/L	3.5–5.0 mmol/L
	Urea	9.8 mmol/L	2.5–7.8 mmol/L
	Creatinine	67 µmol/L	64–104 µmol/L
	eGFR	>90 mL/min/1.73 m²	>60 mL/min/1.73 m²
Coagulation profile	Prothrombin time	11.1 seconds	10–13 seconds
	APTT	21.1 seconds	24–36 seconds
	Fibrinogen	5.1 g/L	2.0–4.0 g/L
Bone and liver profile	Adjusted calcium	2.44 mmol/L	2.20–2.60 mmol/L
	Total protein	52 g/L	60–80 g/L
	Albumin	22 g/L	35–50 g/L
	Globulin	30 g/L	20–35 g/L
	Phosphate	1.20 mmol/L	0.80–1.50 mmol/L
	Bilirubin	5 µmol/L	0–20 µmol/L
	ALP	59 U/L	30–130 U/L
	ALT	15 U/L	0–41 U/L
Urine microscopy	White blood cells	>100 WBC/HPF	0-5 WBC/HPF
	Red blood cells	>100 RBC/HPF	0-3 RBC/HPF
Microbiology	Urine culture	Klebsiella pneumoniae, sensitive to trimethoprim, pivmecillinam; resistant to amoxicillin	-

Computed tomography of the thorax, abdomen, and pelvis revealed bilateral segmental pulmonary emboli involving upper, middle, and lower lobes on the right, and upper and lower lobe segmental branches on the left. Marked intramural gas was noted outlining the bladder walls, consistent with emphysematous cystitis complicated by extraperitoneal bladder perforation, indicative of a severe infective and inflammatory process. Computed tomography of the thorax, abdomen, and pelvis demonstrated marked intramural gas outlining the bladder wall with evidence of bladder perforation, consistent with emphysematous cystitis (Figure 1). Additional coronal and sagittal views further confirmed intramural gas within the bladder wall (Figure 2).

**Figure 1:**
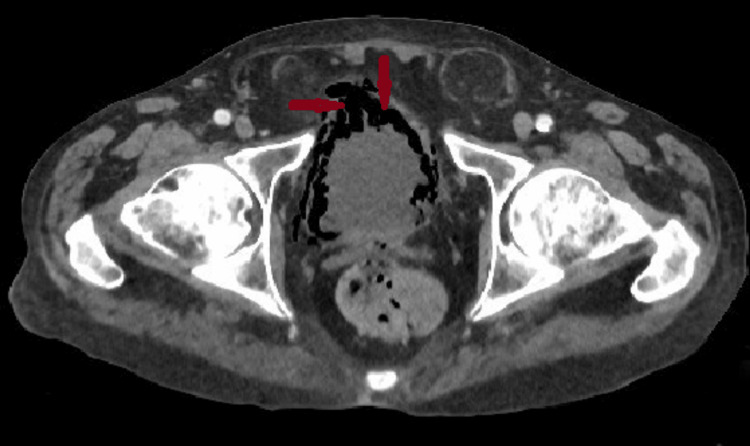
Axial contrast-enhanced abdominopelvic CT image. Computed tomography demonstrated marked intramural gas outlining the bladder wall with evidence of bladder perforation, consistent with emphysematous cystitis (arrows).

**Figure 2:**
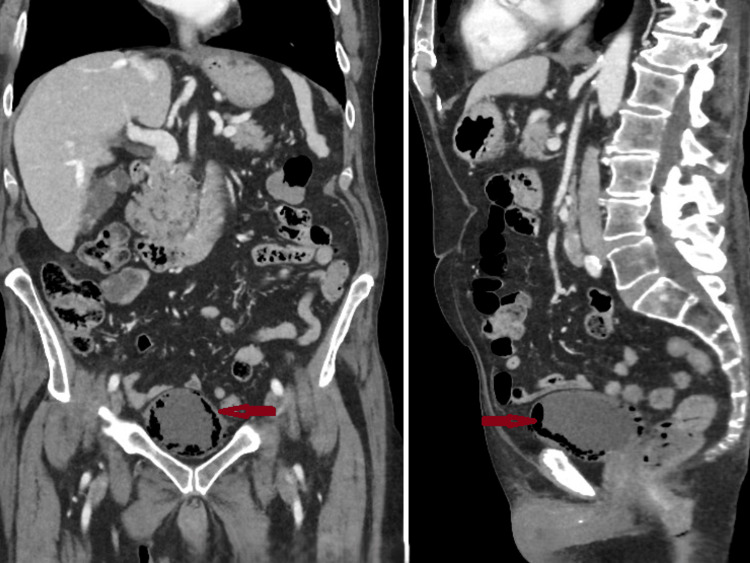
Coronal (A) and sagittal (B) contrast-enhanced CT images of the abdomen and pelvis Computed tomography identified intramural gas (arrows) within the bladder wall, supporting the diagnosis of emphysematous cystitis.

The urology team reviewed the patient and recommended bladder catheterisation using a size 14-fr Foley catheter whilst on antibiotics. Microbiology advised oral pivmecillinam 400 mg three times daily for three weeks, targeting the urinary infection based on urine microscopy, culture, and sensitivity report done two weeks before admission, and apixaban anticoagulation therapy at 10 mg twice daily for seven days, followed by 5 mg twice daily for three months for pulmonary embolism management. 

A repeat contrast-enhanced abdominopelvic CT scan performed four weeks post-admission demonstrated complete resolution of the bladder perforation. Following multidisciplinary review, the patient was discharged with no requirement for further urology follow-up. 

A chronological summary of the patient’s clinical presentation, investigations, and management is outlined in Table 2.

**Table 2:** Timeline of clinical events A chronological summary of the patient’s clinical presentation, investigations, and management is outlined in this table. CRP: C-reactive protein

Timepoint	Events
First clinic visit	Four-week history of malaise with intermittent fatigue, no urinary symptoms, fever or abdominal signs, no infective source identified. CRP 71 mg/L, fluctuation contextually attributed to vasculitic disease, the usual steroid dose was increased.
Second clinic visit (3 weeks later)	Functional decline and weight loss; CRP 59 mg/L, initiated methylprednisolone for presumed vasculitis reactivation, and CT thorax, abdomen, and pelvis requested as outpatient investigation.
Third clinic visit (1 week later)	Screened for possible infection 2 weeks before admission, treated with trimethoprim 200 mg BD for 3 days based on report of urine culture in the absence of gross urinary symptoms.
Hospital admission (6 weeks after initial CT request)	CT report revealed emphysematous cystitis with bladder perforation and bilateral pulmonary embolism, and CRP had risen to 177 mg/L. Patient urgently admitted, Microbiologist advised oral pivmecillinam based on recent urine culture report (*Klebsiella*), bladder catheterisation, and anticoagulation with apixaban commenced for pulmonary embolism.
4 weeks on admission	Repeat CT Thorax, abdomen and pelvis, complete resolution of bladder perforation. CRP normalising.
Discharge	Clinical improvement sustained, discharged with no further urology follow-up required.

## Discussion

Emphysematous cystitis complicated by bladder perforation is a rare but potentially life-threatening form of urinary tract infection characterized by gas accumulation within the bladder wall and lumen. The condition predominantly affects older adults, with a reported median age of approximately 66 years and a female predominance, with a female-to-male ratio of 2:1 [2]. Most reported cases occur in elderly women, and a small proportion of patients, estimated at around 7%, may remain asymptomatic [5].

Emphysematous cystitis is most commonly caused by gas-forming organisms, particularly *Escherichia coli *and *Klebsiella pneumoniae*, accounting for approximately 69% and 29% of cases, respectively [3]. The condition is strongly associated with poorly controlled diabetes mellitus, which is the most frequently reported comorbidity, as well as additional risk factors including immunosuppression, neurogenic bladder, recurrent urinary tract infections, bladder outlet obstruction, and indwelling urinary catheters [5,6].

Clinical presentation is variable and may range from typical lower urinary tract symptoms such as urinary frequency, urgency, dysuria, and haematuria to more severe systemic features. In older adults, presentations are often subtle or atypical, contributing to delayed diagnosis and increased risk of complications. Bladder perforation, although uncommon, represents a serious complication and is associated with significant morbidity. Reported mortality rates range from approximately 7% to 14%, particularly in severe or complicated cases [1].

Prompt diagnosis relies heavily on imaging, with computed tomography representing the most sensitive modality for detecting intramural and intraluminal gas and for assessing the extent of disease [4]. In addition to its diagnostic accuracy, CT provides valuable information regarding bladder morphology, surrounding tissues, and the presence of extraluminal gas or fluid collections. Although bedside ultrasonography may be used as an initial imaging modality in selected cases, its sensitivity is limited, reported at approximately 46.1%, and it should not replace CT when emphysematous cystitis is suspected [5].

Management is primarily conservative, consisting of early targeted antibiotic therapy and bladder decompression via catheterisation. Most patients respond well to medical management alone, with surgical intervention reserved for those with necrotizing infection, bladder perforation, or failure of conservative treatment [5]. The concurrent bilateral pulmonary emboli added complexity to management. These were considered most likely provoked in the context of the patient's acute severe infection, immobility, and frailty. Anticoagulation with apixaban was initiated following careful risk-benefit assessment, particularly given the active infective process and hypoalbuminaemia.

Role of chronic CRP elevation in diagnostic interpretation

This case illustrates a specific diagnostic pitfall in patients with established inflammatory disease. The patient's CRP had fluctuated between 18 and 203 mg/L over 14 months, reflecting chronic vasculitis-related variability and rendering any individual value difficult to attribute to a new infective process. At initial presentation, a CRP of 71 mg/L was consistent with his known inflammatory baseline. Its subsequent decline to 59 mg/L at follow-up appeared to argue against active infection; however, the potent anti-inflammatory effect of ongoing high-dose corticosteroids can attenuate the CRP response in the presence of infection. The later escalation to 99-203 mg/L, occurring during the six weeks between CT request and reporting, was not available to the clinical team at the time and would have materially altered management. In immunosuppressed patients with chronic inflammatory conditions, CRP must be interpreted against that individual's established baseline range rather than as an absolute value. A stable or declining CRP in this context does not exclude a concurrent severe infection.

Long-term high-dose corticosteroid therapy likely contributed to the development of emphysematous cystitis in this patient. Although diabetes mellitus is the most frequently reported risk factor, corticosteroids impair host immune responses, reducing the ability to control urinary pathogens [5,7]. Steroid-induced hyperglycaemia and glycosuria may further promote the growth of gas-forming organisms such as Klebsiella pneumoniae, while suppression of inflammatory responses can mask early symptoms and delay diagnosis [5,8]. Additionally, impaired tissue repair associated with chronic steroid exposure may increase susceptibility to severe infection and complications, including bladder perforation [9]. This case underscores the need for a high index of suspicion for severe urinary tract infections in immunosuppressed older adults presenting with nonspecific symptoms.

Previously reported cases of emphysematous cystitis, including patient demographics, risk factors, causative organisms, symptoms, complications, treatment duration, and management strategies, are summarised in Table 3.

**Table 3:** Selected published cases of emphysematous cystitis. Previously reported cases of emphysematous cystitis, including patient demographics, causative organisms, symptoms, complications, duration of treatment and management strategies.

Study	Age/gender	Risk Factors	Organism	Presenting complaints	Complications	Duration of treatment	Management
Wang et al. (2018) [4]	38/Male	Type 1 diabetes, on haemodialysis	Escherichia coli	Fever, anuria	Emphysematous cystitis with pyelonephritis	14 days	Antibiotics with supportive care
Mamad et al. (2025) [10]	61/Not reported	Type 2 diabetes and end-stage chronic kidney disease on dialysis-dependent	Escherichia coli	Confusional syndrome and shock	Emphysematous cystitis with bladder perforation, complicated by paravesical abscess	Not reported	Antibiotics, exploratory laparotomy, and catheterisation
Al Harrak, et al. (2024) [11]	57/Male	Metastatic prostate cancer	Escherichia coli	Nonspecific symptoms	Emphysematous cystitis	17 days	Urethral catheterisation with antibiotics
Adeyemi, et al. (2020) [12]	76/Male	Poorly controlled diabetes	*Escherichia coli *with *Klebsiella pneumoniae*	Fatigue, forgetfulness, and foul-smelling urine	Emphysematous cystitis	14 days	Urethral catheterisation with antibiotics
Mizuno, et al. (2016) [13]	77/Female	None, gastrectomy and cholecystectomy	Escherichia coli	Acute confusion with urinary retention	bacterial meningitis together with emphysematous cystitis	21 days	Urethral catheterisation with antibiotics
Present case report (2025)	82/Male	Frailty, long-term high-dose steroids	Klebsiella pneumoniae	Fatigue and weight loss	Emphysematous cystitis with bladder perforation	14 days	Urethral catheterisation with antibiotics

## Conclusions

Emphysematous cystitis is a serious and under-recognised threat in frail older adults on long-term immunosuppression. In patients with established inflammatory disease, CRP, whether elevated or declining, cannot reliably distinguish vasculitis-related inflammation from superimposed severe infection, particularly when corticosteroids are simultaneously attenuating both symptoms and the acute-phase response. Although non-diabetic emphysematous cystitis has been reported previously, this case is notable for its presentation in a frail, severely immunosuppressed older adult with minimal urinary symptoms, delayed diagnosis, and bladder perforation

Proactive infection screening by the neurologist proved pivotal in this case. Despite equivocal inflammatory markers and absent urinary symptoms, urine MCS identified *Klebsiella pneumoniae *two weeks before admission. Although the prescribed trimethoprim course was clinically appropriate at the time, it was likely insufficient to eradicate a deeply established intramural infection. Critically, the MCS result directly informed definitive inpatient antibiotic selection, demonstrating the diagnostic value of proactive screening in immunosuppressed patients.

Computed tomography characterisation of the perforation as extraperitoneal was directly decisive in supporting conservative over surgical management, with complete radiological resolution confirmed at four weeks. Explicit perforation typing should be considered a standard component of CT reporting in emphysematous cystitis.

Multidisciplinary involvement across geriatrics, urology, microbiology, and radiology is essential. Pending cross-sectional imaging in immunosuppressed patients should be actively tracked, as radiological deterioration may be the only reliable signal of progression when clinical and biochemical markers are pharmacologically suppressed.
